# Relative age effects in European soccer: their association with contextual factors, impact on youth national teams' performance, and presence at the senior level

**DOI:** 10.3389/fspor.2025.1546978

**Published:** 2025-02-20

**Authors:** Gabriele Morganti, Adam L. Kelly, Alexandra Lascu, Paolo R. Brustio, Elvira Padua, Cristoforo Filetti, Marco Porta, Gianluca Briotti, Bruno Ruscello

**Affiliations:** ^1^ Department of Human Sciences and Promotion of the Quality of Life, San Raffaele Roma Open University, Rome, Italy; ^2^ Research for Athlete and Youth Sport Development (RAYSD) Lab, Faculty of Health, Centre for Life and Sport Sciences (CLaSS), Education and Life Sciences, Birmingham City University, Birmingham, West Midlands, United Kingdom; ^3^ Institute for Health and Sport, Victoria University, Melbourne, VIC, Australia; ^4^ Faculty of Health, Research Institute of Sport and Exercise, University of Canberra, Canberra, ACT, Australia; ^5^ Department of Clinical and Biological Sciences, University of Turin, Turin, Italy; ^6^ Department of Industrial Engineering, Faculty of Engineering, “Tor Vergata” University, Rome, Italy; ^7^ LUISS SportLab, LUISS University, Rome, Italy

**Keywords:** relative age effects, birth advantages, youth soccer, talent identification, selection bias, talent development

## Abstract

**Introduction:**

Soccer systems promote early identification and specialisation practices to satisfy short- and long-term goals—both from sporting performance and financial gains perspectives. In this context, players are (de)selected based on observed performance level and on their ability to conform to given organisational demands, leading to the proliferation of selection biases, such as relative age effects (RAEs), which research has shown to influence both developmental experiences and senior career achievements. Accordingly, this study aims to: (a) investigate the magnitude of RAEs among youth national teams competing in the UEFA U17 European Soccer Championship, and their associations with teams' final ranking, (b) examine whether RAEs magnitude could be linked to cultural and contextual factors, and (c) further explore RAEs at senior level.

**Methods:**

Birth quarter (BQ) distribution of youth national teams (*n* = 80) that competed in one of the five editions (2018, 2019, 2022, 2023, and 2024) of the UEFA U17 European Soccer Championship was recorded. Teams were classified based on their country of origin, RAEs magnitudes, final ranking in the tournament, FIFA points, and national population. Furthermore, the BQ distribution of senior national teams (*n* = 24) that competed at the 2024 UEFA Senior European Soccer Championship was recorded.

**Results:**

Chi-square statistics revealed BQ1s were overrepresented at the U17 level (*p* < 0.001) and showed teams exhibiting low RAEs magnitudes recorded the highest likelihood (odds ratio: 5.67) of finishing the tournament in the bottom four positions. Correlation analyses recorded small to moderate positive correlations between RAEs magnitude and national population (.25) and FIFA points (.33). Further chi-square statistics revealed BQ1s continued to be overrepresented at the senior level, albeit with a weaker effect (*p* < 0.001). However, when the senior BQ distribution was compared to the expected distribution taken from the U17 population, this recorded more BQ4s and fewer BQ1s than expected (*p* < 0.001).

**Discussion:**

The findings presented the focus on youth success, the increased talent pool size, and the competition for selection interact to reiterate RAEs' prevalence in European soccer. Moreover, they highlighted initial RAEs define players' journey within the soccer system, whereby relatively older players remain overrepresented at the senior level, albeit to a weaker and lesser extent.

## Introduction

Talent identification (TID) entails the detection of young players displaying the potential to succeed in the future (1). It occurs as early as the first developmental stages, usually resulting in early entrance to soccer high-performance environments at under nine (i.e., 8–9 years of age) (2). Talent development (TD) aims to provide selected players with optimal learning environments to facilitate and accelerate their progression through to the elite levels of sport (1). Both TID and TD processes in soccer have become critical issues and increasingly professionalised for clubs and national federations. Large sums of money are invested (3) and many figures (i.e., scouts, managers, players' agents, families, and intermediaries) are involved in the process of identifying, selecting, and developing talented youth players (4). This is largely due to the organisational structures, at both federations and club levels, which often employ deterministic models of talent pathways (5). These deterministic models encourage early identification and specialisation practices to facilitate players' skill-acquisition processes and to satisfy academies' short- and long-term goals—both from sporting performance and financial gains perspectives (4). Entrance into such learning environments is characterised by a high level of competitiveness, whereby players are continuously assessed, valued, and ranked. Indeed, institutional (and financial) support is offered only to the few players who have received social recognition and validation of their talent to be noticed and considered for the next developmental stage (6). In line with this, during TID and TD processes, players are (de)selected based on their *observed* performance level and *perceived* potential (7), coupled with their ability to conform to given organisational demands and standards (i.e., meet pre-determined developmental and competitive goals) (8, 9).

However, past studies have highlighted the limitations of selecting a few players based solely on early ability, athleticism, and performance standards. This causes the removal from the system of the many unable to comply with the organisational demands, not considered for further development, who may decide to drop out from the sport in question (10, 11). Further emphasising the potential inefficiencies of early selection, research conducted on analysing players' career trajectories highlighted how, contrary to expectations, the vast majority of early selected players are unable to complete the youth-to-senior transition (12–14). As an example, Höner et al. (15), in their prospective study conducted on German soccer, found that only 0.6% of the U12 players selected for a national training program developed into professional-level soccer players. This suggested that an early entrance (i.e., ≤12 years) into professional soccer academies is not a prerequisite for senior success (16). Indeed, there are multiple pathways to reach the highest level of soccer competition (i.e., playing in the FIFA World Cup), as developmental pathways are shaped by cultural and contextual factors. Even in geographical areas with strict-selection policies, such as Europe, nearly half of the players of the 2022 FIFA Men's World Cup began their professional academy training after the sampling years (i.e., >12 years). In line with this, Boccia et al. (17) revealed how less than 10% of players selected to represent Italy at the U16 level were subsequently able to complete the transition (i.e., playing with the senior national team) and suggested it is only as players get older (i.e., ≥21 years) that their youth performance correlates with their senior performance. Similarly, Brustio et al. (18), investigating career trajectories of U17 players representing the English, French, German, Italian, and Spanish national teams, have shown fewer than 15% of them progressed to their respective senior teams.

Further research on this area has highlighted that an increased level of competition and selection pressures cause soccer systems to select players based on their current level of performance, causing the proliferation of selection biases (19). Relative age effects (RAEs) are the most studied selection biases across the soccer landscape. These arise from the decision of soccer organisations to adopt a cut-off criterion that groups children into (bi)annual age groups. From the very first stages of development, players born at the beginning of the selection year are favoured compared to those born at the end (20). Past research has shown the presence of RAEs in male youth soccer worldwide (21, 22) and indicated that player selection procedures, coupled with increased competition for selection, play an important role in the proliferation of this selection bias (23, 24). Specifically, studies revealed relatively older players (a) are more represented at national and international levels compared to regional and/or recreational (25–27), and (b) are favoured at an increasing level of competition for the few available positions (i.e., larger talent pools), whereby RAEs are less pronounced in smaller soccer nations (i.e., small population, lower soccer culture, or performance levels) (28–30). This body of literature proposed that early born players receive more openings into talent pathways due to age-related differences consisting of more time to practice, compete, and develop (31), and highlighted the importance of considering cultural and contextual factors when analysing RAEs presence and prevalence.

In line with this, research suggested RAEs are more prevalent in performance-oriented contexts when there is a need for competitive advantages (32, 33). Indeed, studies conducted on investigating the presence of RAEs and its correlations with team performance highlighted that selecting players born earlier in the year is an important aspect for successful performance outcomes in youth soccer, as results revealed that older teams record significantly higher points per game [e.g. (33),]. Moreover, in German youth soccer, Augste and Lames (34) found a significant and positive correlation between teams' median birth date and final ranking (i.e., an earlier median birth correlated with a better ranking). Similar results were obtained in Swedish youth soccer, where Söderstrom et al. (35) revealed a correlation between positive match outcomes and the higher presence of early born players. Accordingly, in a system characterised by higher competition pressures, relatively older players are preferred over their younger peers (36, 37), emphasising how coaches (and clubs) are focussed on performance outcomes rather than player development. In line with this, past research has presented that raising awareness about the existence of RAEs and their implications does not contribute to their eradication from youth soccer (38, 39). Nevertheless, from an organisational perspective, studies have proposed systemic interventions like rotating cut-off dates (40, 41), a more flexible chronological approach (42) and grouping teams using the average team age method (33) to pursue fairer youth soccer participation and competition.

Theoretical frameworks explaining the mechanisms of RAEs from a sociocultural perspective presented how such advanced developmental opportunities (i.e., early entrance into a high-performance environment) experienced by early born players may further exacerbate age-related differences (43, 44). More specifically, studies conducted in senior soccer have highlighted that early born players continue to be overrepresented (45–47). For instance, Yagüe et al. (48) showed how RAEs were present in 9 out of 10 of the top-10 European leagues. However, whilst these “knock-on-effects” exist, the strength of them decreases. Indeed, recent research aimed at investigating whether relative age (dis)advantages interact with players' ability to complete the youth-to-senior transition has showed that relatively younger players, once selected, have the greatest likelihood of completing the transition, a phenomenon known as the “underdog hypothesis” (18, 37, 46). As such, this body of knowledge shows multiple interacting effects associated with RAEs within soccer that occur on different timescales of the talent pathway and highlights the need for further research.

Accordingly, this study aims to: (a) investigate the prevalence of RAEs among teams competing at the UEFA U17 European Soccer Championship, by outlining their presence and associations with teams' final ranking in the tournament, (b) examine whether RAEs prevalence among national teams could be linked to cultural and contextual factors, such as FIFA points and national population, and (c) further explore relative age (dis)advantages at senior level. For this reason, this study was divided into two parts. Part 1 explored the birthdates of European youth soccer players who have competed at the UEFA U17 Championship and calculated the RAEs magnitude for each national team competing in the tournament to explore relative age associations with on-field results, FIFA points and national population. Part 2 recorded the birth quarter distribution of European senior soccer players who competed at the UEFA Senior Championship to explore relative age (dis)advantages influence on senior career achievements. For Part 1 of the study, it was hypothesised that RAEs were largely present and would influence teams' final rankings at youth levels. For Part 2 of the study, it was hypothesised an increase in the presence of relatively younger players at the senior level, compared to youth levels.

## Materials and methods

### Subjects

In Part 1 of this study, a total sample of 1,565 male European youth soccer players, who competed at the UEFA U17 male European soccer championship, born between 2001 and 2007 (both years included), was considered for the statistical analyses. To be eligible for inclusion, a player must have played in the UEFA U17 male European soccer championship throughout one of the following editions: 2018, 2019, 2022, 2023, and 2024 seasons. The analysis excluded the 2020 and 2021 seasons because the tournaments were not held due to the COVID-19 pandemic. In Part 2 of this study, a total sample of 624 male European senior soccer players, who competed at the UEFA Senior male European soccer championship, born between 1983 and 2007 (both years included), was considered for the statistical analyses. To be eligible for inclusion, a player must have played at the 2024 edition of the UEFA Senior male European soccer championship. Because all data were freely available from the internet and reported anonymously, no approval by an ethical committee was required.

### Procedures

The data for this study (i.e., players' team selection, birthdates, national teams' final rankings) were publicly available and retrieved online from the Transfermarkt website (Part 1 of the study: https://www.transfermarkt.it/u17-europameisterschaft-2024/startseite/pokalwettbewerb/7E24, accessed on 28th July 2024; Part 2 of the study: https://www.transfermarkt.it/euro-2024/startseite/pokalwettbewerb/EM24, accessed on 10th August 2024). Players were classified based on their birthdate [Birth Quarter 1 (BQ1) = January, February, and March; BQ2 = April, May, and June; BQ3 = July, August, and September; and BQ4 = October, November, and December], cohort of play (Youth or Senior), and respective national team (Youth National Teams included in the study = 80; Senior National Team included in the study = 24). The observed birthdate distribution of each cohort was calculated for each BQ. The observed BQ distribution of the youth cohort was compared to the expected distribution of an assumed equal number of players; whereas in order to gain a full understanding of any age bias effects the observed BQ distribution of the senior cohort was compared to both the uniform distribution and the U17 distribution.

Moreover, to comprehend RAEs influences on youth performance outcomes, for Part 1 of the study, youth national teams were classified based on their RAEs magnitude (i.e., Low RAEs, Medium RAEs, Strong RAEs, and Very Strong RAEs; these were obtained through Cramér's V analysis, further details will be given in the following section), and on their final ranking in the tournament (i.e., Level 1 teams = first four positions; Level 2 teams = from 5th to 8th position; Level 3 teams = from 9th to 12th position; Level 4 teams = from 12th to 16th position). Furthermore, to understand whether national teams' RAEs magnitude values were influenced by cultural and contextual factors, national teams' FIFA points and national population were also collected for each team included in Part 1 of the study. FIFA points were publicly available and retrieved online from the Inside FIFA website (https://inside.fifa.com/fifa-world-ranking/men; accessed on 25th August); whereas nation population were also publicly available and retrieved online from the Wikipedia website (https://it.wikipedia.org/wiki/Pagina_principale; accessed on 5th September).

### Data analysis

In Part 1 of the study, a chi-square goodness-of-fit test (*χ*^2^) was used to compare the observed U17 BQs distribution for the whole sample, for each youth national team, and for each country, to an assumed equal number of players (i.e., 25% for each quartile), as already done in other international RAEs studies (14). Since chi-square statistics cannot reveal the magnitude and the direction of an existing relationship for significant chi-square outputs, effect sizes (Cramér's V) and odds ratios (ORs) were also calculated. Cramér's V were used to classify youth national teams based on their RAEs magnitude and were interpreted as follows: values between 0.120 and 0.278 indicated low RAEs prevalence in the team, 0.279 and 0.340 indicated medium RAEs, 0.341 and 0.410 indicated strong RAEs, and 0.411 or more indicated very strong RAEs (49). The ORs and 95% CIs were used to compare BQs for the achievement of youth European status. These were calculated with the youngest group used as reference (BQ4). CIs including 1 (i.e., 95% CI 0.90–1.10) marked no association. Subsequently, a chi-square test for independence (*χ*^2^) was used to investigate youth national teams' RAEs magnitude (set as the independent variable) influence on final ranking in the tournament (set as the dependent variable). Furthermore, the Pearson correlation coefficient (*r*) was also calculated to explore associations between national teams' RAEs magnitude and their cultural and contextual factors (e.g., national teams' FIFA points and national population). Pearson's *r* values below 0.10 indicated trivial associations, between 0.11 and 0.30 small associations, 0.31 and 0.50 moderate association, and 0.51 or more indicated large association (50).

In Part 2 of the study, a chi-square goodness-of-fit test (*χ*2) was used to compare the observed BQs Senior distribution to both the uniform distribution [i.e., assumed equal number of players (25%) for each quartile], and the U17 distribution.

## Results

**Figure 1:**
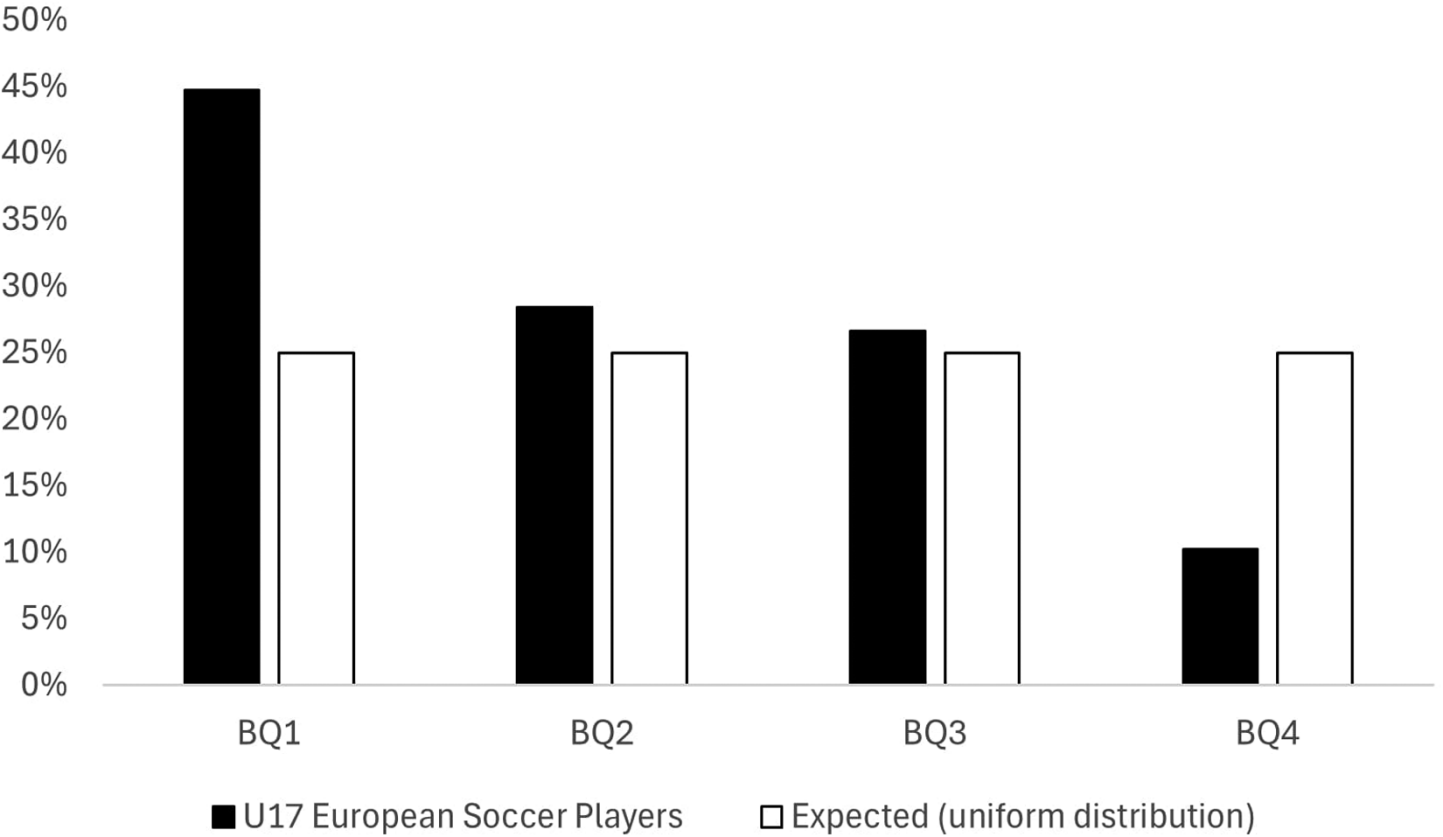
The observed BQs distribution for the U17 European soccer players compared to the expected distribution taken from an assumed equal number of players for each BQ.

The observed BQs distribution for the U17 European soccer players, as well as the expected distribution, are separately displayed in Figure 1. The results revealed a significantly skewed birthdate distribution favouring BQ1 players [*χ*2 (3) = 432; *p* < 0.001; very strong effect size]. The descriptive ORs shown an increased likelihood of players born in BQ1, BQ2, and BQ3 of playing at the U17 European level compared to players born in BQ4 [ORs BQ1 vs. BQ4 (95% CI) = 4.38 (3.52–5.46); BQ2 vs. BQ4 = 2.78 (2.21–3.50); BQ3 vs. BQ4 = 1.63 (1.28–2.07)].

**Figure 2:**
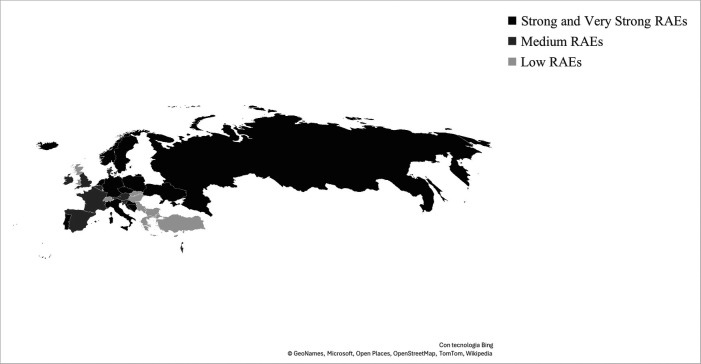
Youth National teams' RAEs magnitudes from a country-level perspective.

Table 1 reports the results from the chi-square goodness-of-fit test conducted from a country-level perspective. Findings confirmed the UEFA U17 European Soccer Championship appears as an early born player affair, as 23 out of 34 countries that have lined up at least one national teams during the investigated editions presented an underrepresentation of relatively younger players and suffered from RAEs (67.6%). More in detail, Bosnia and Herzegovina, Croatia, Czechia, Germany, Iceland, Italy, Netherlands, Norway, Poland, Portugal, Russia, and Ukraine were the countries to select most BQ1s for their UEFA U17 campaigns (strong and very strong RAEs magnitudes; please see Figure 2 for more details on this). Overall, ORs statistics presented BQ4s' decreased likelihood of competing at the U17 highest European level for very strong RAEs magnitudes' countries (ORs ranging from 5.50–14.00), strong RAEs magnitudes' countries (ORs ranging from 2.00–8.00), and medium RAEs magnitudes' countries (ORs ranging from 2.20–6.14).

**Table 1:** Bqs distribution of the Youth European soccer players from a country-level perspective compared to the expected distribution taken from an assumed equal number of players for each BQ.

Youth National Teams	BQ1: % (expected)	BQ2: % (expected)	BQ3: % (expected)	BQ4: % (expected)	*χ2*	*P*	*V*	ORs BQ1 vs. BQ4
Austria	45 (25)	25 (25)	20 (25)	10 (25)	10.4	**0**.**01**	0.29	**4.50** (**1.12–18.13)**
Belgium	42.1 (25)	29.8 (25)	21.1 (25)	7 (25)	14.9	**<0**.**01**	0.29	**6.00** (**1.65–21.76)**
Bosnia and Herzegovina	57.9 (25)	26.3 (25)	5.3 (25)	10.5 (25)	12.8	**<0**.**01**	0.47	5.50 (0.77–39.51)
Bulgaria	30 (25)	20 (25)	35 (25)	15 (25)	2.0	0.57	0.18	3.00 (0.40–22.71)
Croatia	52.5 (25)	17.5 (25)	17.5 (25)	12.5 (25)	16.5	**<0**.**001**	0.37	**4.20** (**1.13–15.59)**
Cyprus	35 (25)	25 (25)	20 (25)	20 (25)	1.2	0.75	0.14	1.75 (0.31–10.02)
Czechia	50 (25)	25 (25)	15 (25)	10 (25)	15.2	**<0**.**01**	0.35	2.00 (0.63–6.38)
Denmark	46.7 (25)	25 (25)	13.3 (25)	15 (25)	16.9	**<0**.**001**	0.30	**3.11** (**1.10–8.78)**
England	45.5 (25)	28.6 (25)	14.3 (25)	11.7 (25)	22.3	**<0**.**001**	0.36	**3.89** (**1.48–10.23)**
France	45.5 (25)	28.6 (25)	14.3 (25)	11.7 (25)	20.3	**<0**.**001**	0.29	**5.33** (**1.83–15.58)**
Germany	46.8 (25)	33.8 (25)	10.4 (25)	9.1 (25)	31.3	**<0**.**001**	0.36	**5.14** (**1.84–14.36)**
Greece	40 (25)	15 (25)	30 (25)	15 (25)	3.6	0.30	0.24	2.67 (0.43–16.39)
Hungary	30.8 (25)	28.2 (25)	25.6 (25)	15.4 (25)	2.1	0.54	0.13	2.00 (0.53–7.50)
Iceland	63.2 (25)	15.8 (25)	10.5 (25)	10.5 (25)	14.9	**<0**.**01**	0.51	6.00 (0.84–42.78)
Ireland	42.4 (25)	30.5 (25)	18.6 (25)	8.5 (25)	15.2	**<0**.**01**	0.29	**5.00** (**1.50–16.62)**
Israel	43.6 (25)	25.6 (25)	17.9 (25)	12.8 (25)	8.4	**0**.**03**	0.26	3.40 (0.89–12.92)
Italy	53.5 (25)	28.3 (25)	12.1 (25)	6.1 (25)	53.4	**<0**.**001**	0.42	**8.53** (**3.21–24.29)**
Luxembourg	38.9 (25)	33.3 (25)	16.7 (25)	11.1 (25)	3.7	0.28	0.26	3.50 (0.45–27.02)
Netherlands	52.5 (25)	27.5 (25)	12.5 (25)	7.5 (25)	39.2	**<0**.**001**	0.40	**7.00** (**2.43–20.13)**
Norway	44.4 (25)	44.4 (25)	11.1 (25)	0 (25)	9	**0**.**02**	0.39	8.00 (0.69–20.13)
Poland	50 (25)	27.6 (25)	15.5 (25)	6.9 (25)	24.3	**<0**.**001**	0.37	**7.25** (**2.03–25.92)**
Portugal	57.5 (25)	27.6 (25)	15.5 (25)	6.9 (25)	69.5	**<0**.**001**	0.48	**11.40** (**3.91–33.21)**
Russia	60 (25)	30 (25)	5 (25)	5 (25)	16.4	**<0**.**001**	0.52	**12** (**1.10–130.59)**
Scotland	28.2 (25)	25.6 (25)	28.2 (25)	17.9 (25)	1.1	0.77	0.09	1.57 (0.43–5.76)
Serbia	38 (25)	22 (25)	13 (25)	14 (25)	9.5	**0**.**02**	0.20	2.14 (0.88–5.22)
Slovakia	25 (25)	40 (25)	20 (25)	15 (25)	2.8	0.42	0.22	1.67 (0.25–11.07)
Slovenia	28.9 (25)	44.7 (25)	13.2 (25)	13.2 (25)	10.4	**0**.**01**	0.30	2.20 (0.55–8.81)
Spain	43.4 (25)	30.3 (25)	19.2 (25)	7.1 (25)	28.6	**<0**.**001**	0.31	**6.14** (**2.32–16.27)**
Sweden	47.4 (25)	28.2 (25)	15.4 (25)	9 (25)	26.9	**<0**.**001**	0.33	**5.29** (**1.90–14.70)**
Switzerland	32.4 (25)	32.4 (25)	16.2 (25)	18.9 (25)	1.5	0.66	0.18	1.71 (0.47–16.31)
Turkiye	36.8 (25)	15.8 (25)	36.8 (25)	10.5 (25)	4.3	0.22	0.27	3.50 (0.46–26.43)
Ukraine	70 (25)	20 (25)	5 (25)	5 (25)	22.8	**<0**.**001**	0.62	**14** (**1.30–150.90)**
Wales	26.3 (25)	23.7 (25)	36.8 (25)	13.2 (25)	5.2	0.15	0.21	2.00 (0.49–8.11)

Bold = statistically significant at <0.05.

When investigating each youth national team participating in the four investigated editions of the UEFA U17 European Soccer Championship, independently of its country, descriptive statistics revealed 28.7% of them exhibited very strong RAEs prevalence (*n* = 23), 26.3% strong RAEs (*n* = 21), 21.2% medium RAEs (*n* = 17), and 23.7% low RAEs (*n* = 19).

**Figure 3:**
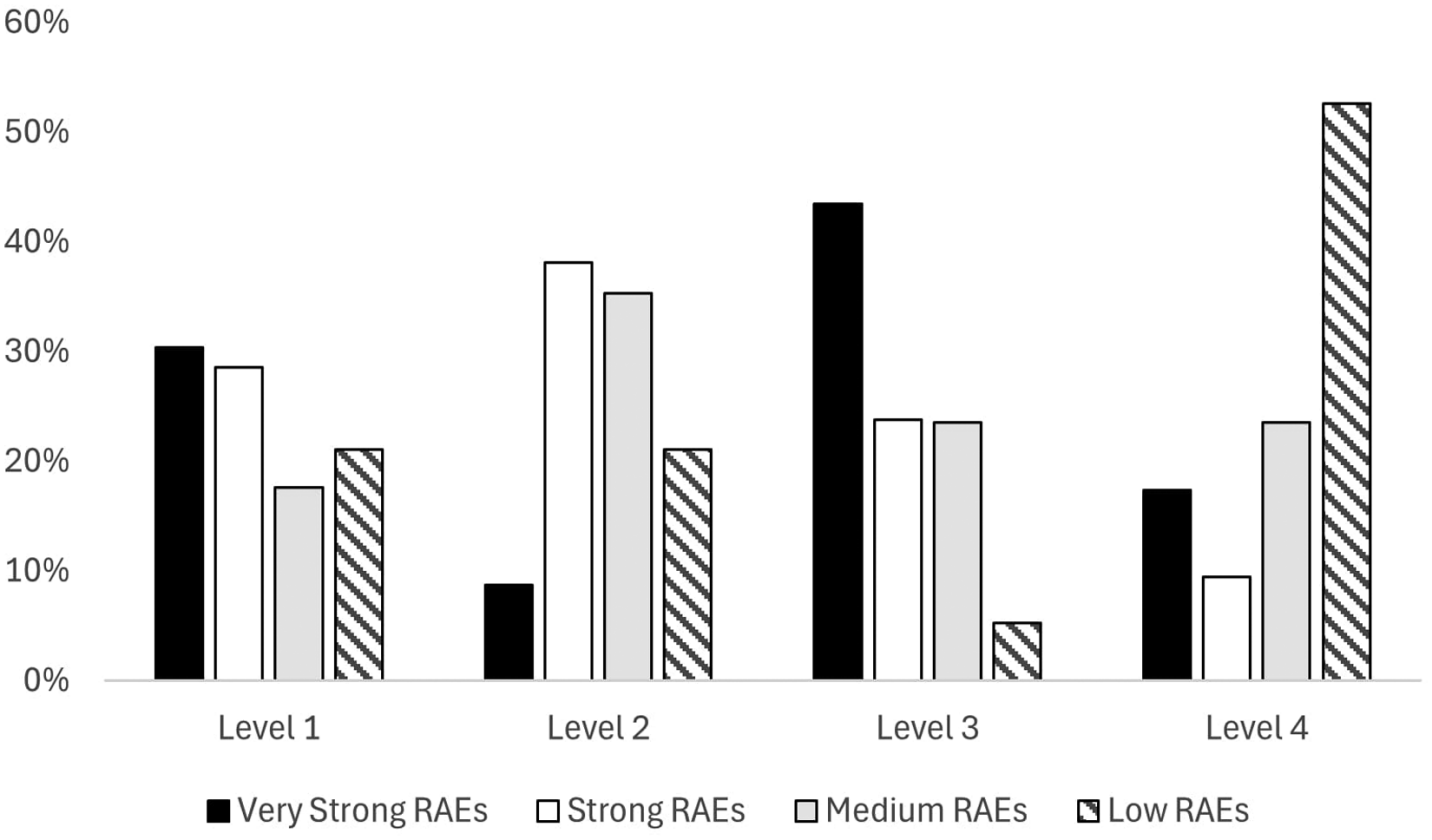
Youth National teams' RAEs magnitudes divided per final ranking.

The chi-square test for independence revealed a significant association between youth national teams' RAEs magnitude and their final ranking in the tournament [*χ*2 (9) = 20.1; *p* = 0.017; very strong effect size]. More in detail, Figure 3 separately displays the distribution of youth national teams' RAEs magnitude divided per final ranking. Teams with very strong and strong RAEs magnitudes recorded the highest proportions of teams finishing in the top four positions (30.4% and 28.6%, respectively). In contrast, national teams with medium and low RAEs magnitudes were more likely to finish in the bottom four positions (23.5% and 52.6%, respectively). In line with this, results from the ORs exhibited low RAEs magnitude teams were 5.67 (1.84–17.5) more likely to rank as Level 4 teams and reported a tendency (not statistically significant) of youth national teams exhibiting very high RAEs magnitude of finishing the championship in the top 4 positions [1.48 (0.50–4.37)].

Regarding the correlation analysis, a small to moderate positive correlation was recorded between national teams' RAEs magnitude and national population [*r* (78) = .25; *p* = 0.02], as well as FIFA points [*r* (78) = .33; *p* < 0.01].

**Figure 4:**
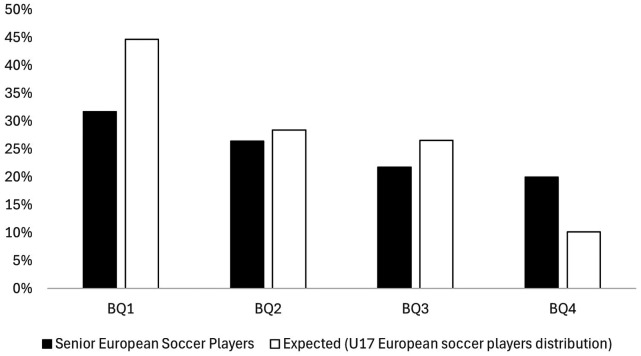
The observed BQs distribution for the Senior European soccer players compared to the expected distribution taken from the U17 cohort.

The observed BQs distribution of the Senior European soccer players, as well as the uniform distribution and the expected distribution obtained from the U17 distribution, are separately displayed in Figure 4. Results revealed early born players continue to overrepresented at senior level, albeit to a lesser and weaker extent [*χ*2 (3) = 20.3; *p* < 0.001; moderate effect size]. Indeed, further chi square analysis revealed a statistically significant differences between the U17 BQ distribution and the Senior BQ distribution [*χ*2 (3) = 93.5; *p* < 0.001; strong effect size], whereby the latter recorded more BQ4s than expected [BQ4 (expected value) = 20% (10.2)], and fewer BQ1s than expected [BQ1 (expected value) = 31% (44.7%), see Table 2 for more detailed information]. In line with this, ORs highlighted how BQ4 players were more likely to be represented at Senior European level than at the U17 level compared to BQ1s, BQ2s, and BQ3s [ORs BQ4 vs. BQ1 (95% CI) = 2.80 (1.96–3.98); BQ4 vs. BQ2 = 2.13 (1.47–3.09); BQ4 vs. BQ3 = 1.50 (1.01–2.23)].

**Table 2:** Bqs distribution of the senior European soccer players compared to the expected distribution taken from the U17 distribution.

Senior European Players	BQ1 (expected)	BQ2 (expected)	BQ3 (expected)	BQ4 (expected)	*χ2*	*P*	*V*
N	198 (279.2)	165 (177.4)	136 (103.7)	125 (63.7)	93.5	**<0** **.** **001**	Strong
%	31.7 (44.7)	26.4 (28.4)	21.8 (16.6)	20 (10.2)			

Bold = statistically significant at <0.05.

## Discussion

This study's aim was threefold: (a) to investigate the prevalence of RAEs among teams competing at the UEFA U17 European Soccer Championship, by outlining their presence and associations with teams' final ranking in the tournament, (b) to examine whether RAEs prevalence among national teams could be linked to cultural and contextual factors, such as FIFA points and national population, and (c) to further explore relative age (dis)advantages at senior level. The results from Part 1 of this study revealed the population of the U17 European soccer players is overrepresented by relatively older players, who are more likely to represent their country at the international youth level. In line with this, 28.7% of youth national teams exhibited very strong RAEs prevalence and recorded the highest proportion of teams finishing the tournament in the top four positions. In contrast, youth national teams with low RAEs prevalence (23.7%) had the highest likelihood of completing the tournament in the bottom positions. Further investigations exploring associations between relative age prevalence and cultural and contextual factors, revealed a moderate association between national teams' RAEs magnitude and their FIFA points. Results from Part 2 of this study presented early born players remained overrepresented at the senior level, albeit with a lower and weaker effect. Indeed, results also showed the BQ distribution of senior European players recorded more BQ4s and fewer BQ1s than expected [BQ4 (expected value) = 20% (10.2); BQ1 (expected value) = 31% (44.7%)].

The results from Part 1 of this study demonstrated how youth players' participation at the UEFA U17 European Soccer Championship is biased toward favouring early born players and confirmed RAEs in male youth soccer are widespread internationally (51), as most of the countries who lined up their youth national team in at least one of the five investigated editions have reported from medium to very strong RAEs magnitudes. Findings are in line with past research conducted at the UEFA youth level (21, 22, 36) and linked to the climate youth soccer systems operate in, which is characterised by financial and results pressures (4). Barraclough et al. (7) have recently highlighted that in soccer, youth players' current performance standard is strongly and positively correlated to coaches' perceptions of their potential and is used to guide selection procedures and to differentiate between player's skill and ability levels (i.e., the formation of elite underage groups). In line with this, past research has shown selection decisions in youth soccer aim to find the most promising young players (2, 52) who align with club's requirements (i.e., required attributes and style of play) (6, 53). However, researchers have already presented that when selection processes are driven by the need to answer to specific functional demands such as the need to build winning age-group teams (54, 55) soccer systems indirectly cause the proliferation of selection biases (i.e., RAEs). This phenomenon is particularly pronounced as born players are favoured due to a combination of age-related differences (31) and sociocultural and environmental factors (20, 42, 56). Specifically, they experience more time to practice, compete, and develop, as well as greater opportunities to build vital cognitive and psychosocial skills (57). This allows them to achieve higher performance standards in the early stages of development which guarantee them more openings into talent pathways, resulting in early access to better training facilities, competent technical staff, and higher competition levels (4, 58). This, in turn, leads to a further rise in performance and soccer-specific skills as well as the opportunity to build important relationships with coaches (i.e., social visibility), resulting to advantages on different timescales (i.e., short- and long-term effects of RAEs) (42).

Part 1 of this study has also investigated the possible relationship between RAEs and cultural and contextual factors. Results recorded positive correlations between national teams' RAEs magnitude and their FIFA points and national population, thus indicating the higher the FIFA points and the national population, the higher the RAEs magnitudes of the respective national team. These findings confirm previous research conducted in this area, as past studies have already presented RAEs presence is more prevalent as levels of competition increase (i.e., competing for selection) (23, 24). More in detail, in Scottish youth soccer, Dugdale et al. (29) found the presence of RAEs differs based on the level of play. The authors found no RAEs at the amateur level, whereas they recorded evident birth asymmetries among youth players competing at higher levels. Similar results were also found in England (59), Portuguese (60), and German (61) youth soccer, where weaker RAEs were found at the lowest level of play. In this study, the higher prevalence of RAEs magnitudes among national teams displaying higher FIFA points may be attributed to the highest level of domestic play, which likely intensity selection pressures and, in turn, contribute to the amplification of RAEs (44). Moreover, research has also presented a higher talent pool size is associated with an increased likelihood of selecting relatively older players over their younger counterparts (62). For example, Figueiredo et al. (60) found that RAEs were prevalent in young Portuguese international soccer players but were not pronounced in young Portuguese international futsal players. In a similar vein, a recent investigation conducted by Bennett et al. (63) on Australian soccer specifically aimed at investigating how the member federation size influences RAEs prevalence, they reported that an increase in 760 affiliated players led to a 1% higher selection probability for those born in the first 6 months of a chronological age group. As such, findings from our study which correlated higher national population to higher RAEs magnitudes may be attributed to the fact that a larger population dispose of a larger talent pool size to select from for their youth national representatives, thus causing increased competition for the few available positions in the line-up and reiterating the presence of relative age (dis)advantages.

The focus put on current performance standards is related to the practical and theoretically well-sounding assumption that the most promising youth players are the ones able to outperform their peers (2, 10, 55). Indeed, on a shorter timeframe, this linear and deterministic approach assures early successes (i.e., players' skill improvement, and rise in performance level), and guarantees an increase in players' values both on and off the field (i.e., matches results and market value) (8, 9, 55). Accordingly, the achievements of youth teams (i.e., national and/or international trophies; players signing professional contracts) are celebrated in newspapers and social media alike, to demonstrate soccer governing bodies are investing for the future, and players are on the right developmental pathway (64–66). However, results from our study showed performance outcomes in international tournaments appear associated with national teams' RAEs magnitude, therefore showing how lining up early born players may be required to finish the championship in the top positions, corroborating previous findings in the area (33–35). These results indicate that by emphasising youth soccer success, practitioners continue to reiterate inequalities in opportunities to develop. This eventually undermines the pool of available talent to select from at the senior level by giving the best developmental and competitive opportunities only to players able to perform at the highest levels, while removing low performers from the system, without considering any possible interindividual variations in players' developmental status and their potential implications for accurate decision-making (6, 10). Indeed, as presented by Fürst (6), athlete selection differs from talent selection as “not everyone who demonstrates potential for future excellence will be selected (or even considered talented) due to factors such as biases and practical constraints, for instance, the availability of players or the coach's limited knowledge about certain individuals” (p.81). In the case of national team selections, practical constraints may be even more amplificated, as head coaches can select a maximum of 20 players for the next matches and/or tournaments (i.e., increased selection competition and pressures), whereby only the ones with the required functional attributes and performance standards will be given the chances to represent their country at the international stage, and this could eventually build on RAEs prevalence. Moreover, as presented by Morganti et al. (67) due to limited social visibility, not all players can be seen and considered by their respective national team head coaches.

Results from Part 2 of the study further explored the complex relationship between relative age (dis)advantages and senior career achievements. These findings confirmed previous research that underlined RAEs at early developmental stages continue to persist and influence performance at both youth and senior stages, although they decline with increasing age (18, 45–47, 59, 68). For example, McAuley and colleagues (45) have recently presented that in Northern Irish soccer, despite no RAEs being found at the senior level, 50% of Northern Irish players selected at the U17 level that were subsequently selected for the senior team were from BQ1, compared to the only 14% born in BQ4, thus revealing possible long-term effects of relative age (dis)advantages, also confirmed in other studies (68, 69). Specifically, Heilmann et al. (68) recorded the presence of RAEs in German third-division professional soccer. The authors revealed these were observed due to the cohort of young players (born after 1998), whose birthdates were significantly skewed toward favouring BQ1s, in contrast, they recorded no asymmetries in the cohort of older players (born before 1998). These results confirm past research, which suggested the beginning of a youth career in soccer is affected by RAEs (70).

However, transient effects of relative age (i.e., decrement of its magnitude at older ages) need to be further investigated as in some cases they appear as the result of RAEs reversal (18, 37, 71, 72). More in detail, Figueiredo et al. (73) explained RAEs as the difference between observed and expected distribution. In the case of senior professional soccer, the expected distribution is represented by the BQ distribution of the younger categories (46). As such, in the context of this study, the U17 European players population represents the expected values. Importantly, when comparing the Senior European BQ distribution to the U17 European BQ distribution, the mitigation of RAEs is explained by the augment in the percentages of players from BQ4. Accordingly, later born players may display the highest likelihood of completing the youth-to-senior transition (18, 46). Researchers attributed several motivations for RAEs reversal, known as the “underdog hypothesis”, whereby relatively younger individuals may improve their psychological, technical, and tactical skills to overcome age disadvantages, ultimately developing the required character to compete at the senior level (74). A recent study conducted by Andronikos et al. (75) which aimed at investigating factors contributing to the youth-to-senior transition, indicated factors such as personal resources (i.e., technical attributes, coping strategies, physical condition, self-expectations) and the ability to think positively in any situation as strong and positive predictors of adjustment to senior sport. In line with this, Bolckmans and colleagues (71) in their retrospective study on youth international Belgian soccer players, reported self-confidence [defined as “showing faith in one's skills, the courage to meet difficult situations, and the pleasure one has in playing soccer” (p.4)] was the personality construct that most defined future career outcomes. Those in BQ4s were more likely to score higher in self-confidence than BQ1s, and recorded the highest proportion of players developing into professionals. More in detail, McCarthy et al. (76) showed that initial age advantages experienced by earlier born players (i.e., more time to practice and compete and cognitive and psychosocial skills), cause low levels of early challenges (i.e., higher performance standards), and act as push factors, pushing them to the next developmental stage. However, the authors reported early advantages correlated to an external focus, whereby these players were motivated by winning, being recognised as talented, and gaining selection for a national program. On the other side of the same coin, relatively younger players, due to age-related differences, experience high levels of early challenges that authors displayed correlated to an internal focus, whereby these players were motivated by enjoyment and personal development. Therefore, these two different pathways may help define and characterise players' journeys within the soccer system. For example, experiencing and overcoming early challenges may help develop the right coping mechanisms for future challenges (76).

The breadth of relative age (dis)advantages means that there is an inherent risk within talent identification and development processes. A risk matrix developed by Baker et al. (10) presented how practitioners' tendency to overlook potential in favour of performance outcomes may cause, on one side, the recurrence of false positive errors, consisting of the promotion of players displaying a high level of performance but low long-term potential. On the other side, such a vision causes the reiteration of false negative errors, demoting players from the talent system when performing below given standards despite high long-term potential. Accordingly, soccer systems invest large sums of money, time, and resources (i.e., personnel and structures) (2, 77) in players who will miss the youth-to-senior transition, as already shown by longitudinal research on players' careers (14, 17). Indeed, a recent study by Barth et al. (78) showed how youth performance can only explain 2.2% of the variance in senior performance. As such, this suggests how celebrating youth success and increasing youth performance levels and standards do not linearly lead to an increase in future senior performance. Therefore, soccer systems should not celebrate early results as they can only explain a little part of the developmental journey and are not correlated to future achievements. Indeed, results from our study suggested early successes are achieved through the reiteration of selection biases derived from athlete selection procedures favoured over talent selection ones (6).

## Practical implications and future directions

Many of the discussion points raised so far are not novel, and yet their continued presence in sporting systems is undeniable. This perpetuation of RAEs comes in many forms, from a focus on athlete selection rather than talent selection, an overemphasis on youth success, and the overshadowing of harm caused by systemic (dis)advantages for athletes (79). In this final section, we aim to cast out a thread that goes beyond the repeated calls for more research, for better sporting systems, and for holistic approaches to talent identification and development. Understandably, these calls are becoming hollow as they echo through a Special Issue featuring 40 years of research on RAEs. To break from this echo chamber, we need to radically consider the role that we play, the *practical implications* for those in a position to make change, and forge a path forward together.

While the depth and breadth of conversation needed to unpack why athlete selection and talent selection are not the same thing is beyond the scope of this paper, it is worth briefly reiterating the complexity of “talent”. Blurry terminology and poor theory/conceptualisation, coupled with flawed evaluation methods due to periods of variation and instability in maturation and development, has weakened the predictive capacity of talent identification and forecasting initiatives (80, 81). Furthermore, the ease and convenience of using athlete selection makes the push for talent selection all the more difficult; even using the word “talent” has become controversial (80). If selection is at the crux of the issue, what would happen if we removed this need to select teams at youth levels?

When we overemphasise the importance of youth performances, it is often for capitalistic gain within the existing system (55): to win tournaments and championships for prestige and money, despite research repeatedly demonstrating that this does little to prepare athletes for future senior performance. If the perceived need to comply with the provision of teams at international tournaments (and to win them) were removed, then a greater focus on the long-term development of talent could be enacted. This deeper, sociocultural pressure through economics and youth tournament success is incredibly difficult to overcome, and it appears that research into its ineffectiveness will not be enough to create a shift in philosophy and perspective.

While sporting systems remain wedded to athlete selection, the magnitude of RAEs may allow for some regulation of the effects within selected teams. Asking youth performance teams to report the magnitude of RAEs as a benchmarking exercise may explicitly call out selection biases in the hopes of counteracting them, although this may not be enough given the long-term effects of RAEs even when they do not appear in senior performance teams [e.g., 45]. There is also a risk that using RAEs magnitude as a target could mean it stops being an effective measure of relative age effects [Goodhart's Law; as cited in Mattson et al. (82)], where teams begin to target athlete selection based on the benchmarking and not talent selection, moving further away from the intentions of the program.

Let us imagine that such a feat has been achieved, that selection for youth performance teams is no longer necessary. How would we “find the best talent” in the pool of participants? A robust approach to holistic, care-full development of young people distributed through a broader network of talent development environments could focus on the development of factors that do contribute to elite senior performance: overcoming challenges, building self-confidence and “personal resources” linked to a successful progression through the pathway. Again, this recommendation has been seen across multiple areas of athlete development and most recently explored as a dual-pathway approach termed by Till and colleagues (83) as “wide and emergent—narrow and focussed”. Not without its challenges, a broader system means even greater difficulty when identifying talent, with a need for strong alignment of what is considered talent across sports, environments and many coaches, creating organised chaos at best (83).

From an organisational perspective, it is worth presenting systemic strategies and proposals that past research has reported and advanced to lower inequalities in selection procedures and opportunities to develop (84). For instance, Boucher and Harley (85) suggested shortening age group categories to 9 months. Hurley et al. (40) proposed the relative age fair cycle system to rotate cut-off dates, whereby players can experience being both the oldest and the youngest in their given cohort throughout their developmental process. Kelly et al. (86) suggested a more flexible chronological approach, which offers relatively older and younger players the opportunities to play up and down their respective age groups (42). Similarly, Helsen et al. (87) introduced a new age-grouping method targeted at levelling the playing field (i.e., mitigating somatic and physical fitness variations in youth soccer), reallocating youth players according to their median birth date calculated between their chronological and estimated developmental birth dates. Moreover, to remove pre-defined selection time points and chronological age groups, Kelly and colleagues (86) proposed the birthday-banding methodology, where young athletes move to the next birthdate group on their birthday. Further research presented that giving additional support to relatively younger players (developmental training camps exclusively opened to later-born players, less emphasis on results, and equal playing time at the earliest developmental stages) coupled with a dynamic grouping strategy (i.e., variating the cut-off date between 1 January and 1 July annually) could correspond to a significant decrease in RAEs presence (88).

Accordingly, investigating youth soccer clubs' RAEs magnitudes through a quantitative approach is needed to continue raising questions and debates on relative age (dis)advantages in a sport system that emphasises selection over development to propose eventual solution mechanisms (79). Moreover, to obtain a more comprehensive understanding of RAEs influence on performance outcomes and career achievements, studies should also focus on players' metrics like playing time (i.e., starting players vs. substitutes), match impact (i.e., goals and assists scored, and match grades), and performance statistics (i.e., physical and technical match outputs). However, deepening our knowledge of RAEs from outside of the system has not been enough to eradicate them, so a greater qualitative exploration of the mechanisms that perpetuate their presence in sporting systems from practitioners' and coaches' perspectives must persist. The entanglement of unique aims related to both youth players' selections and their developmental outcomes via examination of terminology and discourses around talent (identification, selection, and development), with conventional standards and cultural and socio-economic backgrounds (i.e., what entails to be a talented youth player), needs continue investigation to create more equitable talent pathways. Furthermore, considering the limited available studies to explore the effects of possible RAEs solutions, future research should also investigate their eventual positive short- (i.e., increased equality in selection procedures and competition across birth quarters) and long-term (i.e., continued soccer participation and career outcomes) effects.

## Limitations

When interpreting the results of this study, it is important to consider its limitations. First, only being part of the respective U17 or Senior National Team roster was required to be included in this study. However, some players could have played considerably more games than others (e.g., regular starts and substitutes). Accordingly, appearances and/or impact on the UEFA European Championship could be variables included to obtain a greater understanding of how relative age (dis)advantages define selection decisions and national teams' performance outcomes. Second, this study did not consider the duration of youth players' careers and investigated relative age (dis)advantage without considering players' past (i.e., retrospective) and future (i.e., prospective) career trajectories. Involving a longitudinal research design would have contributed to gaining a better insightful knowledge of how RAEs interact with players' progression through the system. Third, playing time and playing positions were not included as variables in this study. Including playing time when studying RAEs in soccer is important to examine the influence of players' participation levels on RAEs outcomes, whereas playing positions would guarantee a better understanding of who is more vulnerable to this selection bias. Fourth, the observed relationships between national teams' RAEs magnitudes and their FIFA points and national population may result from differences in national youth development soccer systems (i.e., promoted practices, pay-to-play model, spatial distribution of sport-specific facilities, regular ways of being and doing things) and soccer popularity.

## Conclusions

The increased adult involvement in youth soccer, coupled with the recent habit of celebrating under-age teams' achievements in newspapers and social media alike, has resulted in professionalised TID and TD practices, indirectly causing the promotion of early identification and specialisation procedures, which often lead to several selection biases (i.e., RAEs), thus calling for further exploration on the area of birth advantages in soccer. This study highlighted the focus on youth results and the competition for selection (i.e., national population and FIFA ranking points) interact with the reiteration of RAEs (dis)advantages in soccer. Specifically, success at the UEFA U17 European Soccer Championship is associated with national teams' RAEs magnitudes. Furthermore, it showed that youth-level RAEs define players' journeys within the soccer system. Considering RAEs resulting from talent identification and development procedures, future research should aim to use a mixed-method approach. Indeed, quantitative studies are required to assess and evaluate relative age (dis)advantages, whereas qualitative studies are needed to comprehend the root causes of RAEs by investigating the terminology and discourses around talent (identification, selection, and development), conventional standards (i.e., what entails to be a talented youth player), and cultural and socio-economic backgrounds.

## Data Availability

The raw data supporting the conclusions of this article will be made available by the authors, without undue reservation.
